# Assessment of Dietary Intake Patterns and Their Correlates among University Students in Lebanon

**DOI:** 10.3389/fpubh.2014.00185

**Published:** 2014-10-21

**Authors:** Pascale Salameh, Lamis Jomaa, Carine Issa, Ghada Farhat, Joseph Salamé, Nina Zeidan, Isabelle Baldi, for the Lebanese National Conference for Health in University Research Group

**Affiliations:** ^1^Clinical and Epidemiological Research Laboratory, Faculty of Pharmacy, Lebanese University, Hadath, Lebanon; ^2^Department of Nutrition and Food Sciences, Faculty of Agricultural and Food Sciences, American University of Beirut, Beirut, Lebanon; ^3^Faculty of Public Health, Lebanese University, Fanar, Lebanon; ^4^Faculty of Health Sciences, University of Balamand, Beirut, Lebanon; ^5^Charité – Universitätsmedizin University Hospital, Berlin, Germany; ^6^Laboratoire Santé Travail Environnement, Université Bordeaux Segalen, Bordeaux, France

**Keywords:** dietary pattern, food categories, gender difference, university students

## Abstract

**Introduction:** Unhealthy dietary habits are major risk factors for chronic diseases, particularly if adopted during early years of adulthood. Limited studies have explored the food consumption patterns among young adults in Lebanon. Our study aimed to examine common dietary patterns and their correlates among a large sample of university student population in Lebanon, focusing on correlation with gender and body mass index (BMI).

**Methods:** A cross-sectional study was carried out on 3384 students, using a proportionate cluster sample of Lebanese students from both public and private universities. A self-administered food frequency questionnaire was used to assess dietary intake of university students. Factor analysis of food items and groups, cluster analysis of dietary patterns, and multivariate regressions were carried out.

**Results:** Three dietary patterns were identified among university youth namely a vegetarian/low calorie dietary pattern (characterized mainly by consumption of plant-based food while avoiding “western” food, composite dishes, and bread); a mixed dietary pattern (characterized by high consumption of plant-based food, followed by composite dishes, bread, and a low consumption of western type food); and finally, a westernized dietary pattern (characterized by high consumption of white bread and western food, and a strong avoidance of plant food and composite dishes). We observed significant differences between males and females in terms of their reported food intake and dietary patterns. Females were particularly more prone to adopt the vegetarian/low calorie diet than males (ORa = 1.69; *p* < 0.001), while males were more likely to adopt a westernized diet (ORa = 1.51; *p* < 0.001), seemingly in private universities (*p* = 0.053). Students with high income and obese students (BMI ≥ 30 kg/m^2^) were more likely to consume vegetarian/low calorie diets (*p* < 0.05).

**Conclusion:** Male university students reported a higher consumption of the westernized dietary pattern as compared to female university students in Lebanon, while the latter reported a higher adoption of a vegetarian diet. Health promotion programs are needed to address the dietary intakes and lifestyle behaviors of young adults in Lebanon to help prevent obesity and other associated comorbidities.

## Introduction

Unh ealthy dietary habits are among the major risk factors for obesity and related chronic diseases, particularly if adopted during early adulthood (1, 2). They are becoming more frequent due to the nutritional transition that is affecting populations across developing countries (3, 4), where traditional healthy diets, including the Mediterranean diet, are being progressively replaced by more westernized dietary patterns (5, 6).

University students seem to be the most affected by this nutrition transition (7, 8); studies from developed countries have shown that young adults leaving their parents and living away from home to attend college experience numerous health-related behavioral changes, including the adoption of unhealthy dietary habits (9–11). These behaviors are mostly attributed to drastic changes in the environment and resources available, frequent exposure to unhealthy foods and habits (12), leading to higher consumption of high caloric snacks, fast foods, and lower consumption of fruits and vegetables, i.e., replacing their consumption of nutrient-dense foods with energy-dense nutrient-poor foods (13); added to this, skipping meals may also become more frequent (14).

Studies in the Middle East show that adolescents and adults eating behaviors are adversely being influenced by the changing environmental-factors leading to alarming rates of overweight and obesity and higher metabolic risk factors causing diabetes, hypertension, and other chronic diseases (15, 16). In Lebanon, a small country in the Middle East, the prevalence of overweight and obese adolescents and young adults was reported to be as high as 21 and 11%, respectively, significantly higher than those reported 10 years before (17). Previous studies were conducted among university students in Lebanon; however, these focused mostly on one or two private universities, showing that the nutrition transition was associated with higher rates of obesity among youth (18, 19).

Gender differences in eating and weight-related behaviors have been reported in the scientific literature from developed nations such as Canada (20), France (21), and the United States (22). To our knowledge, few similar studies were carried out in Middle Eastern countries that explore gender differences among university students. In Lebanon, a previous study conducted on students from one university showed that females had healthier eating habits than males and lower rates of obesity (18). Among adults of Beirut, women had also healthier dietary intakes (23). Moreover, in an earlier study conducted on a sample of all university students in Lebanon exploring various health risk behaviors, our research group showed how obesity prevalence differed between males and females (24). Given these findings, the objective of this analysis was to define the dietary patterns of university students in Lebanon, exploring their respective correlates, focusing primarily on gender and weight status.

## Materials and Methods

### General study design

A cross-sectional study was conducted between 2010 and 2011, using a proportionate cluster sample of students from 16 private universities and the only public university in Lebanon. Based on data of student population across various universities from the Center for Pedagogic Researches in Lebanon (25), a proportionate sample of 3000 students was targeted to allow for adequate power for bivariate and multivariate analyses to be carried out.

The study was waived from IRB approval at the Lebanese University; however, researchers and field worker conducted the study according to the research ethics guidelines laid down in the Declaration of Helsinki (26).

Verbal informed consent was also obtained from all subjects prior to participating in the study and completing the self-administered questionnaire.

### Procedure

Random selection of university students was not possible at the various institutions in Lebanon due to administrative challenges, thus a convenient sample of students was recruited for this study. A trained field worker approached students outside their classrooms during break hours and explained the study objectives. Students who expressed interest and provided their oral consent were handed a self-administered anonymous questionnaire that included questions related to sociodemographic, anthropometric, dietary, and lifestyle behaviors. On average, the questionnaire was completed by participants within approximately 20 min. At the end of the process, the completed questionnaires were placed in closed boxes and sent for data entry. During the data collection process, the anonymity of the students was guaranteed. Out of 4900 distributed questionnaires, 3307 (67.5%) were returned to the field worker, thus the sample size needed for sufficient power to conduct the analyses was met. Further methodological details were presented by authors elsewhere (24).

### Dietary intake assessment

The self-administered questionnaire used in this study included numerous questions related to the socio-demographic background of university students and a short food frequency questionnaire (FFQ) to assess the usual dietary intake of youth. The FFQ was composed of 16 semi-quantitative questions covering different food categories (including the five basic food categories typically consumed by the Lebanese population) (27). The FFQ used in this study was adapted from the questionnaire earlier administered in the Lebanese population (27) and the CDC Global School Health Survey (28); the finally used items were vegetables and salads, fruits, olive oil, grains (lentils, peas), fish and seafood, meats (including cooked meats, poultry, ham, and hotdog), white bread and derivatives, brown bread and derivatives, rice and pasta, sweets (cake, ice cream, chocolate, …), carbonated beverages, fruit juices, hot beverages (coffee, tea, Nescafe, hot chocolate, or milk), cooked vegetables (mainly for composite dishes), fast food [hamburger, pizza, Lebanese pizza (known as Man’ouche with thyme or cheese or yogurt based kechek), and sandwiches], and fried potatoes and chips. We omitted to ask questions about eggs and dairy products as separate items, because they would have been confusing to the students to record in the FFQ given that these food items are frequently consumed in Lebanon within composite dishes (eggs, cheese, and yogurt within cooked dishes), fast food meals (in sandwiches and Lebanese pizzas), or as part of hot beverages (hot chocolate or Nescafe, and hot milk). The FFQ asked how often each food item, group, or beverage was usually consumed with five possible answers for each of the food categories: (1) never, (2) two times or less per week, (3) three to six times per week, (4) at least one time per day, and (5) at all meals. These five response categories were later merged into four categories for analysis purposes, namely: (1) never, (2) once or twice per week (3) three to six times per week, and (4) consumption on daily basis.

### Anthropometric data

Students involved in this study self-reported their weights and heights; however, measurements were conducted by a trained field worker on a subsample of individuals (*N* = 618) using standardized techniques and calibrated scales. A comparison of self-reported versus measured anthropometrics allowed us to calculate equations for corrected reported weights and heights for males and females. The following equations were used for measuring corrected weights and heights: for males [corrected weight = (1.003*reported weight) and corrected height = (0.959*reported height) + 7.59] and for females [corrected weight = (0.942*reported weight) + 3.14 and corrected height = (0.943*reported height) + 9.42].

A corrected body mass index (BMI) was subsequently calculated as corrected weight in kilograms over corrected height squared (in square meter). According to the International Classification of adult weight to height status (i.e., underweight, overweight, and obese), BMI values were classified into four categories for individuals 20 years of age or older: underweight (BMI ≤18.5 kg/m^2^), normal weight (BMI between 18.5 and 24.9 kg/m^2^), overweight (BMI between 25 and 29.9 kg/m^2^), and obese (≥30 kg/m^2^) (29); the method recommended by Cole and collaborators was used (30).

### Other demographic and lifestyle variables

Income level of each student was assessed using the reported household monthly income divided by the number of individuals per household as a surrogate measure. The obtained number was subsequently divided into quartiles of income level, according to which individuals were classified.

In order to assess the physical activity level of students, a questionnaire was used to calculate leisure time physical activity on the basis of mean metabolic equivalents (MET) for reported activities and their frequency and duration in MET – minutes per week; a higher score indicated greater activity (31). Students were asked to report about their habitual leisure time physical activities; these included recreational activities, such as bicycling (MET = 8), basketball playing (MET = 8), and walking for exercise (MET = 4) as well as more structured lessons, such as swimming (MET = 6), dancing (MET = 6.5), and stretching (MET = 2.5). Each student had a physical activity score that was computed by multiplying an estimate of the MET for each reported activity by the weekly frequency with which it was performed and an overall average weekly score was calculated as MET*times per week. Furthermore, time spent on each activity was multiplied by the MET value of the activity. The resulting MET-min products were summed to produce an index of weekly physical activity, expressing the amount of energy per kilograms body weight expended during the week (31).

### Data analysis

Statistical analyses were performed using the Statistical Package for Social Sciences (version 16.0, SPSS, Inc). Student’s *t*-tests were conducted to examine differences in height, weight, and BMI between males and females. Chi-square analyses were used to compare BMI categories distributions, income level quartiles, university types, field of studies, and consumption frequencies of the different food categories between males and females and some nominal variables distributions between clusters.

### Identifying dietary patterns among university students

In nutritional epidemiology, various statistical methods are used to derive common eating patterns among specific populations; factorial and cluster analyses are two of the most common methods. Both methods allow for empirical derivation of eating and dietary patterns: factor analysis derives patterns based on intercorrelations between food items/groups, whereas cluster analysis depends on individual differences in mean intakes when reducing data into patterns (32). In our study, both methods were used: factorial analysis allowed for identifying food group patterns based on intercorrelations between these food components and cluster analysis allowed for grouping individuals within our sample into mutually exclusive groups based on their adherence to these food group patterns. Studies using both empirical methods allow for exploring correlations between derived dietary patterns and various health outcomes (33, 34). The procedure of each is explained in details below:

First, an exploratory factor analysis was performed to identify patterns of food categories consumed by university students from our sample, i.e., to look at food items that were consumed at the same frequency by individuals. After ensuring sample adequacy with the Kaiser–Meyer–Olkin (KMO) index and Bartlett’s Chi-square test of sphericity, factors of food categories consumption were extracted using the principal component analysis and using a promax rotation. Factors with Eigenvalues higher than one were retained; confirmation of adequacy with a Scree plot was performed and interpretability of the results was taken into account. Items with factor loading ≥0.4 were considered as belonging to a factor. Reliability analysis was performed by Cronbach’s alpha values for factors and the total scale.

Second, a cluster analysis was performed with the identified factor scores reflecting patterns of consumption of food categories using the K-mean method to identify dietary patterns consumed by study participants. This method allowed study participants to be grouped into non-overlapping mutually exclusive clusters reflecting their dietary patterns. Analysis allowed for 30 iterations centering results on zero and convergence was only reached using a three clusters structure, i.e., thus, three different dietary patterns.

### Associations between dietary patterns and participant characteristics

Association between socio-demographic characteristics of study participants and the derived dietary patterns were evaluated, using both bivariate and multivariate analyses. For the latter analysis, we carried out a multinomial backward logistic regression, using the full model to show the effect of all independent variables: the dependent variable was the dietary pattern; the major independent variables were gender and BMI, whereas age, university type, income level, and physical activity were all taken as covariates. Adjusted odds ratio (ORa) were calculated, after ensuring model adequacy to data.

## Results

### Characteristics of the subjects

A total of 3307 university students with complete data were included in the analysis; 60% were females (*N* = 1969) and the remaining 40% were males (*N* = 1332). The average age of participants was 20 years, ranging between 17 and 37 years. Differences were detected between male and female university students with respect to their reported income status, distribution between public and private universities, and across various fields of studies. A higher percentage of males were in private universities and reported higher income levels compared to females. Furthermore, a higher percentage of males were classified as overweight or obese, based on the corrected BMI, compared to female university students in our sample (38 versus 10%, respectively, *p* < 0.001) (see Table 1).

**Table 1:** **Sociodemographic and anthropometric measurements of university students in the total sample, and by gender (*N* = 3307)**.

Variable	Total (*N* = 3307)	Males (*N* = 1332)	Females (*N* = 1969)	*p*-Value
			
		Mean (SD)		
Age in years	20.37 (1.86)	20.66 (2.0)	20.18 (1.7)	<0.001
Height (cm)	169.82 (9.84)	178.3 (7.4)	163.9 (6.5)	<0.001
Weight (kg)	65.88 (15.57)	78.2 (15.1)	57.4 (8.8)	<0.001
Physical activity (MET-min/week)	1006.72 (1474.16)	1593.0 (1868.5)	639.9 (1002.3)	<0.001
BMI (kg/m^2^)	22.71 (4.34)	24.6 (4.5)	21.4 (3.7)	<0.001

		***N* (%)**	

BMI categories
Underweight (<18.5 kg/m^2^)	297 (9.0%)	34 (2.6%)	263 (13.9%)	<0.001
Normal (18.5–24.9 kg/m^2^)	2219 (67.1%)	778 (59.4%)	1441 (76.2%)	
Overweight (25–29.9 kg/m^2^)	547 (16.5%)	387 (29.5%)	160 (8.5%)	
Obese (≥30 kg/m^2^)	139 (4.2%)	111 (8.5%)	28 (1.5%)	
Income level				<0.001
Quartile 1	673 (20.4%)	239 (19.6%)	604 (33.6%)	
Quartile 2	741 (22.4%)	215 (17.7%)	409 (22.8%)	
Quartile 3	635 (19.2%)	392 (32.2%)	441 (24.6%)	
Quartile 4	766 (23.2%)	371 (30.5%)	341 (19.0%)	
Public university	1668 (50.4%)	491 (36.9%)	1175 (59.7%)	<0.001
Private universities	1639 (49.6%)	841 (63.1%)	794 (40.3%)	
Field of studies				<0.001
Sciences and informatics	665 (20.1%)	333 (25.3%)	332 (16.9%)	
Health sciences	1088 (32.9%)	210 (15.9%)	876 (44.6%)	
Humanities and laws	283 (8.6%)	73 (5.5%)	210 (10.7%)	
Business and economics	733 (22.2%)	394 (29.9%)	335 (17.1%)	
Engineering	274 (8.3%)	219 (16.6%)	55 (2.8%)	
Arts	243 (7.3%)	89 (6.8%)	154 (7.8%)	

### Dietary Intake of University students based on Gender

Based on the semi-quantitative FFQ, significant differences were observed between male and female university students with respect to their consumption of individual food categories regularly consumed by the Lebanese population (14 out of the 16 food items or groups). Males consumed more fish, white bread, rice and pasta, carbonated beverages, fruit juice, fast food, and fried potatoes and chips than females (*p* ≤ 0.001), while females consumed more brown bread, meat, grains, olive oil, fruits, and raw and cooked vegetables (*p* ≤ 0.001). Only hot beverages and sweets were equally consumed by males and females (see Table 2).

**Table 2:** **Dietary intake of university students as assessed by a food frequency questionnaire, in total study sample and by gender**.

Food category	Total *N* (%)	Males *N* (%)	Females *N* (%)	Chi-square	*p*-Value
Vegetables and salads				31.85	<0.001
Never	53 (1.8)	28 (2.4)	25 (1.4)	
≤2 times/week	627 (20.8)	293 (25.2)	334 (18.0)	
3–6 times/week	770 (25.6)	298 (25.6)	472 (25.5)	
Daily	1563 (51.9)	543 (46.7)	1020 (55.1)	
Fruits				17.62	<0.001
Never	73 (2.4)	37 (3.2)	36 (2.0)	
≤2 times/week	555 (18.5)	240 (20.7)	315 (17.1)	
3–6 times/week	688 (22.9)	282 (24.3)	406 (22.0)	
Daily	1691 (56.2)	602 (51.9)	1089 (59.0)	
Olive oil in food				16.02	0.001
Never	87 (2.9)	25 (2.2)	62 (3.4)	
≤2 times/week	601 (20.0)	245 (21.1)	356 (19.3)	
3–6 times/week	761 (25.3)	329 (28.3)	432 (23.4)	
Daily	1560 (51.8)	562 (48.4)	998 (54.0)	
Grains (lentils, peas.)				15.80	0.001
Never	144 (4.8)	57 (4.9)	87 (4.7)	
≤2 times/week	1424 (47.6)	497 (43.1)	927 (50.4)	
3–6 times/week	1071 (35.8)	445 (38.6)	626 (34.0)	
Daily	354 (11.8)	154 (13.4)	200 (10.9)	
Fish and seafood				73.12	<0.001
Never	305 (10.2)	107 (9.3)	198 (10.7)	
≤2 times/week	2053 (68.6)	718 (62.5)	1335 (72.4)	
3–6 times/week	449 (15.0)	205 (17.9)	244 (13.2)	
Daily	184 (6.2)	118 (10.3)	66 (3.6)	
Meat, hotdog, ham				99.62	<0.001
Never	243 (8.1)	155 (13.4)	88 (4.8)	
≤2 times/week	446 (14.9)	191 (16.6)	255 (13.9)	
3–6 times/week	1259 (42.1)	491 (42.6)	768 (41.7)	
Daily	1046 (34.9)	316 (27.4)	730 (39.7)	
White bread				18.91	<0.001
Never	222 (7.4)	63 (5.5)	159 (8.6)	
≤2 times/week	300 (10.0)	96 (8.3)	204 (11.1)	
3–6 times/week	263 (8.8)	113 (9.8)	150 (8.1)	
Daily	2210 (73.8)	881 (76.4)	1329 (72.1)	
Brown bread				23.05	<0.001
Never	1545 (51.7)	649 (56.4)	896 (48.7)	
≤2 times/week	643 (21.5)	228 (19.8)	415 (22.6)	
3–6 times/week	241 (8.1)	98 (8.5)	143 (7.8)	
Daily	561 (18.8)	176 (15.3)	385 (20.9)	
Rice and pasta				19.81	<0.001
Never	86 (2.9)	42 (3.6)	44 (2.4)	
≤2 times/week	1619 (54.2)	614 (53.3)	1005 (54.7)	
3–6 times/week	926 (31.0)	326 (28.3)	600 (62.6)	
Daily	358 (12.0)	169 (14.7)	189 (10.3)	
Sweets (cake, ice cream, chocolate, etc)				5.56	0.135
Never	72 (2.4)	26 (2.3)	46 (2.5)	
≤2 times/week	876 (29.5)	315 (27.5)	561 (30.7)	
3–6 times/week	758 (25.5)	315 (27.5)	443 (24.2)	
Daily	1267 (42.6)	489 (42.7)	778 (42.6)	
Carbonated beverages				49.42	<0.001
Never	347 (11.7)	103 (9.0)	244 (13.4)	
≤2 times/week	845 (28.5)	268 (23.4)	577 (31.6)	
3–6 times/week	623 (21.0)	257 (22.4)	366 (20.1)	
Daily	1155 (38.9)	518 (45.2)	637 (34.9)	
Fruit juices				38.10	<0.001
Never	321 (10.8)	161 (14.0)	160 (8.8)	
≤2 times/week	1164 (39.1)	391 (34.1)	773 (42.3)	
3–6 times/week	600 (20.2)	216 (18.8)	384 (21.0)	
Daily	890 (29.9)	379 (33.0)	511 (28.0)	
Hot beverage (coffee, tea, nescafe, etc)				4.68	0.196
Never	264 (8.9)	100 (8.7)	164 (9.0)	
≤2 times/week	577 (19.4)	245 (21.3)	332 (18.2)	
3–6 times/week	414 (13.9)	160 (13.9)	254 (13.9)	
Daily	1718 (57.8)	643 (56.0)	1075 (58.9)	
Cooked vegetables				18.60	<0.001
Never	571 (19.2)	265 (23.1)	306 (16.7)	
≤2 times/week	1431 (48.1)	522 (45.5)	909 (49.8)	
3–6 times/week	646 (21.7)	236 (20.6)	410 (22.4)	
Daily	327 (11.0)	125 (10.9)	202 (11.1)	
Fast food (hamburger, pizza, etc)				99.43	<0.001
Never	198 (6.7)	57 (5.0)	141 (7.7)	
≤2 times/week	1742 (58.7)	571 (49.9)	1171 (64.2)	
3–6 times/week	662 (22.3)	312 (27.2)	350 (19.2)	
Daily	367 (12.4)	205 (17.9)	162 (8.9)	
Fried potatoes, chips				7.00	0.072
Never	157 (5.3)	62 (5.4)	95 (5.2)	
≤2 times/week	1094 (36.8)	399 (34.7)	695 (38.2)	
3–6 times/week	836 (28.1)	316 (27.5)	520 (28.6)	
Daily	883 (29.7)	372 (32.4)	511 (28.1)		

### Food categories consumption patterns

Major factors were extracted using factor analysis of the 16 food categories in the administered FFQ; these factors reflected food categories that are consumed with similar frequencies at the individual level. Kaiser–Meyer–Olken value was 0.751 (*p* < 0.001 for Bartlett’s test of sphericity), denoting the sample adequacy for the analysis. All communalities were higher than 0.3, except for fruit juice, which was subsequently removed from the factor as it did not load adequately on any of the extracted factors. Four factors were then extracted, explaining together 49.31% of the total variance:

- Factor 1 showed high loadings of fried potatoes and chips, fast foods, carbonated beverages, and desserts with slightly lower but still a positive loading of hot beverages such as coffee, tea, and Nescafe; this factor was termed “western type foods.”
- Factor 2 showed high positive loadings of raw vegetables and salads, fruits, and olive oil; it was named “plant-based foods.”
- Factor 3 had high positive loadings of rice and pasta, cooked vegetables, grains, fish and seafood, and a negative loading of meats; these were deemed to represent “composite dishes” that were consumed by non-meat eaters.
- Factor 4 was characterized by high positive loading on white bread and high negative loading on brown bread; it clearly showed a preference for white bread consumption among study participants (Table 3).

**Table 3:** **Pattern loading of the four major factor solutions after promax rotation**.

Food item/food category	Factor 1 Western type foods	Factor 2 Plant foods	Factor 3 Composite dishes	Factor 4 Bread
Carbonated beverages	0.756			
Fried potatoes and chips	0.710			
Fast food (pizza, hamburger)	0.663			
Hot beverages (coffee, tea, Nescafe)	0.520			
Desserts	0.451			
Fruits		0.799		
Vegetables and salads		0.773		
Olive oil in food		0.558		
Rice and pasta			0.721	
Grains/lentils, peas			0.579	
Fish and seafood			0.576	
Cooked vegetables			0.505	
Meat, hot dog, luncheon meats			−0.431	
White bread				0.797
Brown bread				−0.734

Moreover, the reliability analysis of the food items gave a moderate value of Cronbach’s alpha (0.463), showing the need for factors segregation. Thus, for the factors described above, reliability was 0.621 for western type foods; 0.597 for plant foods; 0.403 for composite dishes (0.567 without meat); and −1.082 for bread, respectively. In the latter case, the Cronbach alpha was negative due to a negative average covariance among items, and the correlation coefficient between brown and white bread was *r* = −0.352 (*p* < 0.001).

### Dietary patterns of university students

A cluster analysis based on the four factors derived three mutually exclusive clusters, which form 26.8, 35.4, and 37.8%, of all participants respectively. As shown in Table 4, the three clusters were labeled as:

- the “vegetarian/low calorie” dietary pattern (cluster 1 with *N* = 788): as it had a strong inverse correlation with factors 1 and 4 (western type foods and white bread, respectively), and a weak but positive correlation with factor 2 (plant foods),
- the “mixed” dietary pattern (cluster 2, *N* = 1042): had the highest scores for factor 2 (plant foods), followed by factor 3 (composite dishes), and factor 4 (white bread), with a low correlation with factor 1 (western type foods),
- and the “westernized” dietary pattern (cluster 3, *N* = 1112) with the strongest association with factors 1 and 4 (western type food and white bread, respectively), and an inverse correlation with factors 2 and 3 (plant foods and composite dishes, respectively).

**Table 4:** **Classification of university students in the study sample by cluster analysis using food categories factor scoring**.

	Cluster 1 *N* = 788 (26.8%) Vegetarian/low calorie diet	Cluster 2 *N* = 1042 (35.4%) Mixed diet	Cluster 3 *N* = 1112 (37.8%) Westernized diet
Factor 1: western food	−0.74	0.14	0.40
Factor 2: plant food	0.20	0.77	− 0.86
Factor 3: composite dishes	−0.20	0.56	− 0.38
Factor 4: bread	−1.16	0.38	0.47

### Characteristics of study subjects according to dietary patterns

Correlations between subjects’ characteristics and their dietary patterns were presented in Table 5. Subjects following a westernized diet included individuals with a higher weight and height, but not a higher BMI. We observed that the vegetarian/low calorie and the mixed dietary patterns were more adopted by females, whereas the westernized dietary pattern was more common among males (*p* < 0.001). Lower income level students and those from the Lebanese public university were more likely to consume a mixed dietary pattern, while those of higher income level and in private universities were more likely to consume either a vegetarian/low calorie or a westernized dietary pattern (*p* < 0.05). Furthermore, 40% of students (*N* = 418) who follow the westernized dietary pattern corresponded to individuals who reported doing no sports at all whereas the 2nd largest group among westernized dietary pattern consumers (16.7%, *n* = 175) reported doing highest level of physical activities, while students following the vegetarian/low calorie dietary pattern were the most to report low to moderate physical activity levels compared to students adopting the two other dietary patterns (*p* = 0.004).

**Table 5:** **Characteristics of university students in the study sample according to the three identified dietary patterns**.

Variables	Vegetarian/low	Mixed	Westernized	*p*-Value
Age (years) mean (SD)	20.51 (1.95)	20.31 (1.78)	20.33 (1.78)	0.066
Weight (kg) mean (SD)	64.39 (14.39)	64.79 (14.91)	66.95 (16.88)	0.001^a^
Height (cm) mean (SD)	168.06 (9.25)	169.37 (9.96)	170.73 (9.94)	<0.001^b^
BMI (kg/m^2^) mean (SD)	22.67 (4.08)	22.50 (4.58)	22.81 (4.51)	0.281
BMI classes *N* (%)
Underweight (<18.5 kg/m^2^)	60 (8.4%)	105 (10.4%)	97 (9.1%)	0.743
Normal (18.5–24.9 kg/m^2^)	507 (70.8%)	709 (70.2%)	738 (69.3%)	
Overweight (25–29.9 kg/m^2^)	116 (16.2%)	156 (15.4%)	180 (16.9%)	
Obese (≥30 kg/m^2^)	33 (4.6%)	40 (4.0%)	50 (4.7%)	
Gender *N* (%)
Male	182 (29.3%)	414 (36.8%)	504 (45.2%)	<0.001
Female	440 (70.7%)	712 (63.2%)	610 (54.8%)	
Income level quartile *N* (%)
Quartile 1	124 (23.0%)	256 (25.8%)	232 (23.7%)	0.029
Quartile 2	135 (25.0%)	279 (28.1%)	240 (24.5%)	
Quartile 3	110 (20.4%)	218 (21.9%)	241 (24.6%)	
Quartile 4	170 (31.5%)	241 (24.2%)	266 (27.2%)	
University type *N* (%)
Public	333 (53.4%)	667 (59.2%)	590 (53.0%)	0.006
Private	291 (46.6%)	460 (40.8%)	524 (47.0%)	
Physical activity level^c^ *N* (%)
No sports at all	204 (35.4%)	372 (36.4%)	418 (40.0%)	0.004
Low (≤624)	111 (19.3%)	192 (18.8%)	152 (14.5%)	
Moderate (624–1200)	98 (17.0%)	164 (16.0%)	132 (12.6%)	
High (1200–2160)	91 (15.8%)	157 (15.3%)	169 (16.2%)	
Highest (>2160)	72 (12.5%)	138 (13.5%)	175 (16.7%)	

*^a^Westernized diet is significantly different from vegetarian diet and from mixed diet with *post hoc* Bonferoni test*.

*^b^Significant difference between all three diet types with *post hoc* Bonferoni test*.

*^c^Calculated according to quartiles of Metabolic Equivalent (MET) multiplied by the weekly frequency and duration of physical activity*.

### Correlates of dietary patterns

Multivariate analyses were conducted to explore the correlates of dietary intake patterns of university youth, where the mixed dietary pattern was taken as a reference. For the vegetarian/low calorie dietary pattern, we found that females were particularly more likely to report adopting this pattern compared to males (ORa = 1.62; *p* < 0.001). Similarly, students who adopted this dietary pattern where from the highest income level compared to the lowest income level (ORa = 1.45; *p* = 0.021) and had the highest BMI (obese category) versus lowest BMI (underweight category) (ORa = 2.80; *p* = 0.004). Age, physical activity level, and type of university were not found to be significant correlates of the vegetarian/low calorie dietary pattern.

With regard to the correlates of the westernized dietary pattern, we observed that females were less likely to adopt a westernized diet compared to males (ORa = 0.64; *p* < 0.001). For physical activity level, students reporting low to moderate physical activity levels (first and second quartile) were less likely to adopt a westernized dietary pattern compared with those who reported no physical activity [ORa = 0.75 (056–1.00) and ORa = 0.63 (0.47–0.86), respectively]. Age, income, and type of university were not found to be significant correlates for adopting the westernized dietary pattern among the study population (Table 6).

**Table 6:** **Correlates of dietary patters among university students: multivariate analysis**.

Correlates^a^	Unadjusted OR	ORa	95% CI of the ORa	*p*-Value
**Vegetarian/low calories diet versus mixed diet^b^**				
Older age	1.04	1.04	0.98–1.11	0.168
Income level				0.012
Quartile 1 (reference)	1.00			
Quartile 2	0.95	0.93	0.69–1.26	0.630
Quartile 3	1.01	0.94	0.68–1.31	0.721
Quartile 4	1.41	1.45	1.06–1.99	0.021
Body mass index				0.035
Underweight (reference)	1.00			
Normal weight	1.25	1.41	0.96–2.07	0.076
Overweight	1.30	1.45	0.90–2.32	0.136
Obese	1.44	2.80	1.40–5.61	0.004
Private versus public university	1.18	1.23	0.96–1.57	0.099
Higher physical activity level				0.651
No activity (reference)	1.00			
Quartile 1	1.13	1.21	0.88–1.64	0.237
Quartile 2	1.11	1.19	0.86–1.64	0.304
Quartile 3	1.12	1.05	0.76–1.47	0.759
Quartile 4	1.03	1.23	0.86–1.75	0.260
Female versus male gender	1.36	1.62	1.24–2.12	<0.001
**Westernized diet versus mixed diet^c^**				
Older Age	0.78	0.98	0.93–1.04	0.534
Income level				0.468
Quartile 1 (reference)	1.0			
Quartile 2	0.95	0.90	0.68–1.17	0.422
Quartile 3	1.21	1.06	0.80–1.41	0.693
Quartile 4	1.23	1.12	0.84–1.49	0.458
Body mass index				0.616
Underweight (reference)	1.00			
Normal weight	1.13	1.08	0.78–1.51	0.643
Overweight	1.25	0.96	0.63–1.45	0.835
Obese	1.35	1.36	0.73–2.53	0.335
Private versus public university	1.22	1.15	0.92–1.43	0.214
Higher physical activity level				0.038
No activity (reference)	1.00			
Quartile 1 Low physical activity	0.73	0.75	0.56–1.00	0.046
Quartile 2 moderate Physical activity	0.74	0.63	0.47–0.86	0.003
Quartile 3 high physical activity	0.96	0.82	0.61–1.09	0.171
Quartile 4 very high physical activity	1.19	0.86	0.63–1.18	0.359
Female versus male gender	0.69	0.64	0.50–0.80	<0.001

*^a^Physical activity level, body mass index class, and income level are categorical variables; age is a continuous variable*.

*^b^*N* = 1392; Nagelkerke *R* Square = 0.036; Hosmer-Lemeshow test *p* = 0.041*.

*^c^*N* = 1708; Nagelkerke *R* Square = 0.031; Hosmer-Lemeshow test *p* = 0.363*.

## Discussion

In this cross-sectional study, we present findings on the main food categories and dietary patterns adopted by a large sample of university youth in Lebanon. We highlight differences in dietary intakes among university students and explore correlates of dietary patterns, focusing primarily on gender and BMI status. Four main food categories consumed by the study participants were derived, namely western type foods including fried potatoes, chips, fast foods, carbonated beverages, and desserts; plant-based foods including raw vegetables, salads, fruits, and olive oil; composite dishes including grains (rice, pasta, white bread), cooked vegetables, fish, and seafood, and a low intake of red meat.

University students’ consumption of these food categories was further explored allowing for dividing the study participants into three groups referring to three dietary patterns adopted based on the consumption of the four derived food categories. The first vegetarian/low calorie dietary pattern was adopted by more than a quarter of the study participants (26.8%, *N* = 788) and it was characterized by the low consumption of white bread, western type foods, and a higher consumption of plant-based foods only. The mixed dietary pattern was adopted by slightly greater than a third of the university students (*N* = 1042) and was characterized by the high consumption of plant-based foods, followed by composite dishes, and bread as well as a low consumption of western type foods. Whereas the westernized dietary pattern was adopted by the largest group of students in our sample (38%, *N* = 1112) and was characterized by the high consumption of white bread and western foods such as fried potatoes and chips, fast foods, carbonated beverages and desserts, and the strong avoidance of plant-based foods and composite dishes.

Identified dietary patterns in our study were relatively similar to those reported in other studies on the adult population in Lebanon (35, 36). According to a cross-sectional study conducted on a large national sample of Lebanese adults aged between 20 and 55 years old, four dietary patterns were identified; these were mainly the western, prudent, and traditional Lebanese, as well as fish and alcohol dietary patterns. Another case–control study conducted by the same research group on type II diabetic patients and their age and gender-matched controls showed that Lebanese adults adopted four similar patterns: refined grains and cereals, traditional Lebanese, fast food, and meat and alcohol (36). Furthermore, similar patterns were found among adolescents in other countries, where “vegetable,” “fruit,” “sweet/salty snack foods,” and “starchy foods” were reported in the US (37), whereas in Brazil “western,” “traditional,” and “mixed” (38) diets were found. These differences in identified patterns between different studies and settings may be due to numerous environmental-factors including cultural cuisine variations, the availability, affordability, and access to certain types of foods in addition to the nutrition transition status of the countries, the Mediterranean location, and the changes that may affect the nutrition environment that young adults live within their respective settings (39, 40). Other individual-level parameters can also contribute to the differences in dietary patterns including age, gender, health status, food preferences, and the nutrition and health awareness knowledge of the various studied populations (39, 40). In our study, only a select of these individual-level variables were explored as possible correlates of the dietary patterns adopted by university students. The dietary patterns we found were adopted differently by university students.

### Gender and BMI differences

Our study showed that a higher percentage of males were overweight and obese compared to females, while a higher percentage of females were underweight. These results were comparable to those reported among similar university student populations in Lebanon (18, 41), and in other countries (42–45): studies have shown that young female adults are more concerned about their weight status and body image than males, and may adopt various restrictive behaviors to limit their caloric intake and avoid weight gain (41, 42). This was further proven in our study where women adopted vegetarian/low caloric dietary patterns more commonly than men (70 versus 30%), whereas, the westernized diet was particularly common for males compared to females (50% more than females).

As reported in some studies (46, 47), the adoption of a westernized diet was not associated with the BMI status of the young adult population in our study even after adjusting for other correlates in the multivariate analyses. However, there was a significant association between BMI status and the adoption of a vegetarian/low caloric diet, adjusting for other covariates: obese students adopted the vegetarian/low caloric diet more than the mixed dietary pattern compared to underweight students (adjusted OR = 2.8, CI = 1.4–5.6, *p* = 0.004). There are several possible explanations as to why obese students reported food intake that reflects low caloric/vegetarian dietary pattern: obese students may be attempting to limit their dietary and caloric intake as a method to lose weight and similar behaviors have been depicted in the literature (41). Another explanation might be the fact that this result has been influenced by a possible underreporting of dietary intake by obese students (48). Thus, these explanations need to be interpreted with caution since there are other variables that were not considered in this analysis and that may affect the association between the adoption of dietary patterns and BMI status. These variables may include the nutrition knowledge and health awareness of participating students, their possible use of weight-loss medications and supplements that may affect their appetite, their food environment as well as their household food security status that may affect their food choices.

### Physical activity

With respect to the level of physical activity of students and as reported in the literature (47, 49), we found that males had significantly higher levels of physical activity through leisure time activities compared to females. Furthermore, a significant association was observed between physical activity and dietary patterns that remained even after adjusting for other covariates. Students with low to moderate physical activity levels were less likely to adopt a westernized dietary pattern compared to a mixed dietary pattern. However, this was not the case with those with highest levels of physical activity. It is worth noting that students who reported limited to no physical activity on a weekly basis were more likely to be adopting the westernized dietary pattern rich in energy-dense, nutrient-poor foods. This increases our concern that university students with sedentary behaviors are consuming unhealthful dietary patterns that increases their risk of weight gain leading to obesity and its various comorbidities during their young adulthood years. The link between a lifestyle that combines the high consumption of westernized fast food diets and sedentary behavior with increased obesity is well-established in the scientific literature (50).

### Socio-economic status

The association of dietary patterns with income level showed that students with low income level were more likely to adopt a mixed dietary pattern that is considered more traditional, while the highest income level students adopted the vegetarian/low caloric diets (plant-based food consumption and low intake of composite dishes, western foods or breads), even after adjusting for other covariates. This was further demonstrated with the higher odds of adopting a vegetarian/low caloric dietary pattern among private university students compared to public university students. Similar findings regarding the association between high socio-economic status and fruit and vegetable consumption have been reported in the literature (51). These results are in line with those published in previous studies in Lebanon that were conducted on smaller sample sizes of university students (41, 52, 53), school adolescent populations (45, 53), and in other countries witnessing the nutritional transition (42, 54, 55).

Several limitations could, however, be stated for this study: reporting bias is possible given that food consumption frequencies and the weight and height measurements were self-reported by respondents. Additional differential misreporting may be possible across socio-demographic groups (56, 57) and BMI classes (58). Food consumption may be differentially reported by males and females; there is a well-established evidence of underreporting of dietary intakes among females and over-reporting of dietary intakes among males, which may lead to an additional reporting bias (57, 58). The cross-sectional nature of the study also precludes causality judgment between dietary patterns and BMI. The relationship between dietary patterns and the nutritional status of university youth in Lebanon, in addition to other health and nutritional parameters, remains to be established by appropriate prospective studies (59). The nature of the non-random sample in our study may also introduce a selection bias; self-selected individuals may not represent the whole university students’ population (59). Even more caution should be exerted in extrapolating the results to Lebanese youth not enrolled in universities; the latter are expected to present more unhealthy nutritional habits than the university population.

Moreover, we may suggest a validation of the FFQ among young adults from the Lebanese population and the use of portion sizes and servings in addition to food consumption frequencies for more precise results, since our semi-quantitative FFQ did not allow us to estimate portion sizes and caloric intake. Furthermore, the collection of dietary intake by trained researchers with strong nutrition and dietetics background can further improve the accuracy of collected data. Further studies that take into account the abovementioned weak points are recommended. Despite these limitations, we still think that the high level of unhealthy dietary patterns, including the adoption of the westernized diet, by our study population is worth exploring further to identify determinants of food consumption patterns and the associations with obesity, cardiometabolic risk factors among other health outcomes.

## Conclusion

In conclusion, we found that dietary patterns differed among university students; males, particularly in private universities, were more prone to adopt a westernized diet, while females were more prone to follow vegetarian/low calorie diets. Health promotion programs and evidence-based educational interventions are needed to promote healthy eating and active lifestyle behaviors among young adults in Lebanon, including university student population, in an effort to limit obesity-related comorbidities during their adulthood years. Furthermore, given the importance of exploring dietary patterns rather than the intake of individual nutrients and foods in relation to health, further studies are needed to explore determinants of dietary patterns among various populations, and more importantly, the association between identified dietary patterns and health outcomes, including obesity and chronic diseases.

## Author Contributions

Pascale Salameh designed the study and drafted the manuscript; Lamis Jomaa carried out the analysis and assisted in drafting and reviewing the manuscript; Ghada Farhat, Joseph Salamé, and Nina Zeidan contributed to the discussion, reviewed the final manuscript and gave their consent; Isabelle Baldi was the project principal investigator.

## Conflict of Interest Statement

The authors have no conflict of interest to declare. The Lebanese University ethical committee waived the need for ethical clearance due to the observational nature of the study and the non-traceability of individuals’ information.
